# What is the point of pseudokinases?

**DOI:** 10.7554/eLife.07771

**Published:** 2015-05-06

**Authors:** Saravanan Raju, Andrey S Shaw

**Affiliations:** Department of Pathology and Immunology, Washington University School of Medicine, St. Louis, United States; Department of Pathology and Immunology, Washington University School of Medicine, St. Louis, United Statesshaw@pathology.wustl.edu

**Keywords:** kinase complex, extracellular protein phosphorylation, secretory pathway phosphorylation, pseudokinase, tooth enamel formation, human, mouse

## Abstract

The interaction between an active kinase and an ‘inactive’ pseudokinase provides clues about how these enzymes were regulated in the past, and how this regulation has evolved.

**Related research article** Cui J, Xiao J, Tagliabracci VS, Wen J, Rahdar M, Dixon JE. 2015. A secretory kinase complex regulates extracellular protein phosphorylation. *eLife* **4**:e06120. doi: 10.7554/eLife.06120

**Image** The pseudokinase Fam20A has a glutamine (Q) instead of the conserved glutamic acid (E) that forms part of a salt-bridge found in all active kinases

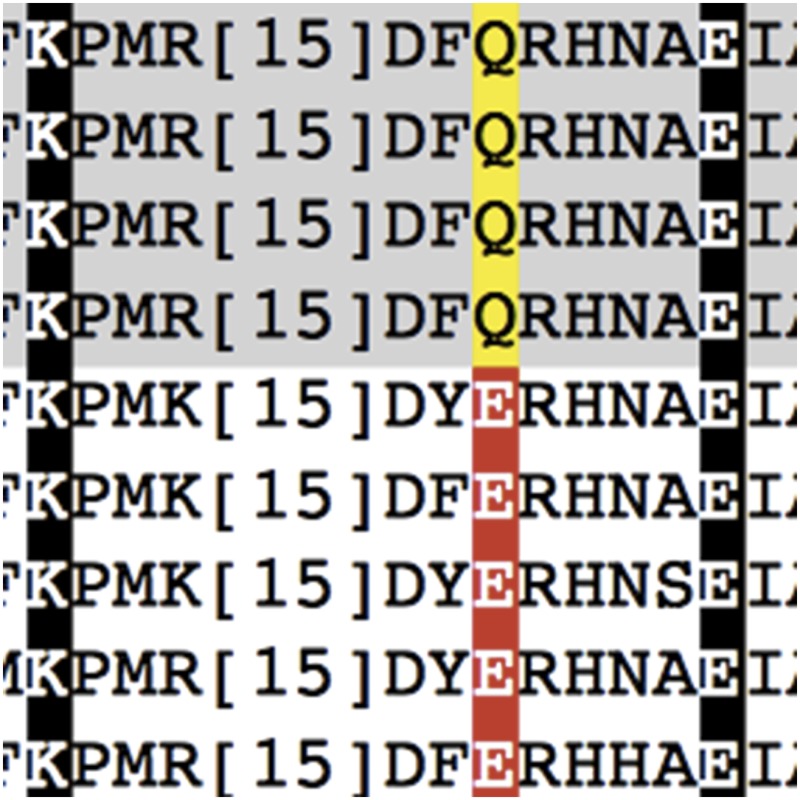

Protein kinases are enzymes that transfer a phosphate group from a molecule of ATP onto a protein or other substrate. This activity, known as ‘phosphorylation’, is important for a wide range of cellular processes. Over the last decade, as we have learned more about how these enzymes work, it has become clear that about 10% of the proteins that had been classified as protein kinases lack some of the key features that are required for kinase activity. These ‘inactive enzymes’, called pseudokinases, have evolved along side their enzymatically active counterparts. This implies that they still have a role to play in nature. Recent data suggest that one of these roles is to regulate the activity of other kinases (Rajakulendran and Sicheri, 2010; Shaw et al., 2014).

While most attention has focused on the role of protein phosphorylation inside cells, the large number of secreted proteins that are phosphorylated suggests that there are extracellular kinases. Jack Dixon of the University of California, San Diego (UCSD) and colleagues had previously carried out a search for secreted kinases and found a small family of ancient and atypical kinases that are responsible for a significant proportion of phosphorylation events that occur outside cells (Tagliabracci et al., 2012). Now, in *eLife*, Dixon and colleagues at UCSD—including Jixin Cui as the first author—provide further support for the idea that pseudokinases continue to carry out an important role, and provide clues to the evolutionary origin of pseudokinases.

Mutations in some these kinases are implicated in several disorders that affect bone and teeth (Simpson et al., 2007; O'Sullivan et al., 2011). For example, mutations in the gene for one of the family members, a kinase called Fam20C, results in the often fatal disorder known as Raine Syndrome. Mutations in the gene for the related Fam20A result in a distinct disorder known as Amelogenesis Imperfecta (which causes weakened and discolored teeth).

At first glance, the differences between these diseases could suggest that these two kinases might be regulated in different ways or target different substrates. But Dixon, Cui and colleagues report something more unexpected. Fam20A is a pseudokinase that controls the kinase activity of Fam20C (Cui et al., 2015).

On its own, Fam20C is a weak kinase. Fam20A, on the other hand, has no measurable kinase activity (even though it can bind ATP); and Dixon, Cui and colleagues explain why Fam20A is a pseudokinase rather than a true kinase. One of the most conserved features in all active kinases is a salt-bridge that forms between a conserved glutamic acid and a lysine. This glutamic acid is missing in Fam20A, which contains a glutamine in its place instead. Notably, this glutamine has also been conserved in Fam20A throughout evolution. When Dixon, Cui and colleagues replaced the glutamic acid in the kinase Fam20C with a glutamine, it significantly reduced the enzyme's activity. Moreover, swapping the glutamine in Fam20A for a glutamic acid turned this pseudokinase into an active kinase.

Dixon, Cui and colleagues then removed the gene for Fam20A in cells and found that the phosphorylation of tooth enamel proteins was significantly diminished. This was the case even through the Fam20C kinase was still present. Deletion of the gene for Fam20C completely abolished enamel protein phosphorylation. Together these findings suggested that the full kinase activity of Fam20C requires Fam20A. In vitro studies then confirmed that the addition of both the kinase and the psuedokinase are required for optimal kinase activity. Both Fam20A and Fam20C can each bind to themselves to form homodimers in solution. They can also bind to each other to form heterodimers. It is likely that the active form of the enzyme is a four-subunit complex as experiments showed that a mixture of Fam20A and Fam20C was a tetramer.

The exact mechanism by which Fam20A promotes the kinase activity of Fam20C is not yet clear. The interactive surfaces seen in the crystal structure of Fam20C could provide clues (Figure 1; Xiao et al., 2013), but a three-dimensional structure of the Fam20C/Fam20A heterodimer will be required for us to understand why it is strongly active while the Fam20C homodimer is not.

**Figure 1.**
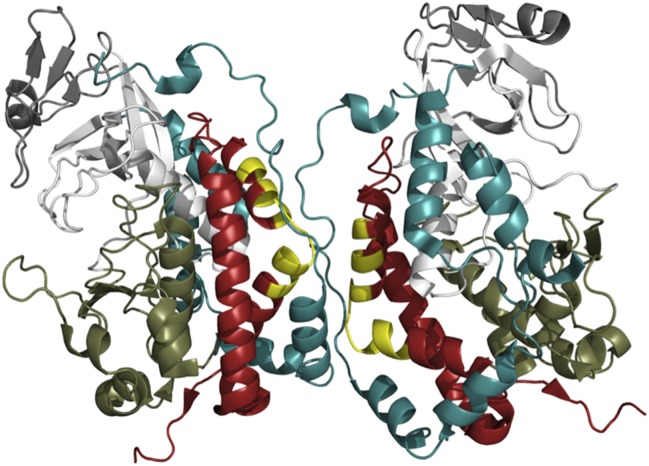
The crystal structure of Fam20C shows a potential dimer interface. Fam20C has an extension to its C-terminus (red); and when two Fac20C enzymes interact in a dimer, a part of this extension from one enzyme directly interfaces with the other enzyme (the interfacing portions are shown in yellow). Fam20C’s N-terminal extension (teal) also interacts with its C-terminal extension, and this interaction could represent the allosteric interface between Fam20C and the pseudokinase Fam20A. This suggests that activation of Fam20C might require the interaction with Fam20A to move these extensions out of way so that the kinase domains (the lower kinase lobes are in olive green, the upper lobes are in white) can directly interact.

FIGURE CREDIT: IMAGE COURTESY OF ALEXANDR P KORNEV (UCSD) AND SUSAN S TAYLOR (UCSD)

Work on other pseudokinase–kinase pairs suggests that the role of pseudokinase binding is to change the shape of the bona fide kinase in the pair into its active conformation (Jura et al., 2009; Zeqiraj et al., 2009; Hu et al., 2013). This kind of regulation is known as ‘allosteric activation’. In the classical model of kinase activation, phosphorylation of a part of a protein kinase called the activation loop triggers its active conformation (Kornev et al., 2008). The fact that Fam20C kinase does not have an activation loop that can be phosphorylated suggests that it is only regulated by its interaction with Fam20A.

The findings of Dixon, Cui and colleagues provide new insights into the evolutionary origin of pseudokinases. While Fam20C had weak kinase activity by itself in vitro, it was only after it was mixed with Fam20A that its true kinase activity was revealed. Fam20A and Fam20C are ancient kinases related to kinases in bacteria and slime molds. The fact that they don't use activation loop phosphorylation for regulation suggests that allosteric regulation (by pseudokinase/kinase heterodimerization) may be one of the earliest methods of kinase regulation. Only later, when the role of the activation loops evolved, did cross-phosphorylation (so-called ‘trans-phosphorylation’) become the dominant mechanism of activation. However, the continued presence of so many pseudokinases in modern organisms suggests that allosteric activation is still an important way that kinases are regulated. In addition, the existence of ‘inactive’ versions of other enzymes, like pseudophosphatases, suggests that dimerization and allosteric regulation could play a significant role in regulation of other enzymatic activities.
